# Development of reverse genetics systems and investigation of host response antagonism and reassortment potential for Cache Valley and Kairi viruses, two emerging orthobunyaviruses of the Americas

**DOI:** 10.1371/journal.pntd.0006884

**Published:** 2018-10-29

**Authors:** James I. Dunlop, Agnieszka M. Szemiel, Aitor Navarro, Gavin S. Wilkie, Lily Tong, Sejal Modha, Daniel Mair, Vattipally B. Sreenu, Ana Da Silva Filipe, Ping Li, Yan-Jang S. Huang, Benjamin Brennan, Joseph Hughes, Dana L. Vanlandingham, Stephen Higgs, Richard M. Elliott, Alain Kohl

**Affiliations:** 1 MRC-University of Glasgow Centre for Virus Research, Glasgow, Scotland, United Kingdom; 2 Department of Diagnostic Medicine/Pathobiology, College of Veterinary Medicine, Kansas State University, Manhattan, Kansas, United States of America; 3 Biosecurity Research Institute, Kansas State University, Manhattan, Kansas, United States of America; Colorado State University, UNITED STATES

## Abstract

Orthobunyaviruses such as Cache Valley virus (CVV) and Kairi virus (KRIV) are important animal pathogens. Periodic outbreaks of CVV have resulted in the significant loss of lambs on North American farms, whilst KRIV has mainly been detected in South and Central America with little overlap in geographical range. Vaccines or treatments for these viruses are unavailable. One approach to develop novel vaccine candidates is based on the use of reverse genetics to produce attenuated viruses that elicit immune responses but cannot revert to full virulence. The full genomes of both viruses were sequenced to obtain up to date genome sequence information. Following sequencing, minigenome systems and reverse genetics systems for both CVV and KRIV were developed. Both CVV and KRIV showed a wide *in vitro* cell host range, with BHK-21 cells a suitable host cell line for virus propagation and titration. To develop attenuated viruses, the open reading frames of the NSs proteins were disrupted. The recombinant viruses with no NSs protein expression induced the production of type I interferon (IFN), indicating that for both viruses NSs functions as an IFN antagonist and that such attenuated viruses could form the basis for attenuated viral vaccines. To assess the potential for reassortment between CVV and KRIV, which could be relevant during vaccination campaigns in areas of overlap, we attempted to produce M segment reassortants by reverse genetics. We were unable to obtain such viruses, suggesting that it is an unlikely event.

## Author summary

Cache Valley and Kairi viruses (CVV and KRIV; *Peribunyaviridae*, *Orthobunyavirus*) are important animal pathogens of the Americas. In this study we developed reverse genetics systems to study and manipulate viral genomes of both viruses. Viral genomes were mutated to prevent the expression of the NSs protein, a key virulence factor and antagonist of the type 1 interferon (IFN) system. Replication studies in IFN producing cell lines showed slower growth of the NSs-deletion carrying viruses compared to wild type virus. In contrast, in IFN-deficient cell lines growth of both viral types was comparable, highlighting the role of NSs as an IFN antagonist in both CVV and KRIV. We also demonstrated using genetic studies that CVV and KRIV are unlikely to combine by reassortment to form novel viruses at least for one combination tested here, and propose that such recombinant viruses would be suitable live attenuated vaccine candidates.

## Introduction

The bunyaviruses are a large grouping of animal and plant-infecting viruses with a segmented, negative-stranded genome. The order *Bunyavirales* was recently proposed to include ‘bunyavirus like’ viruses that could not be assigned to the previous 5 genera (https://talk.ictvonline.org/taxonomy/) [1]. This has resulted in the following new families: *Feraviridae*, *Fimoviridae*, *Hantaviridae*, *Jonviridae*, *Nairoviridae*, *Phasmaviridae*, *Phenuiviridae* and *Tospoviridae*; the remaining *Peribunyaviridae* family, previously called *Bunyaviridae*, contains the previous *Orthobunyavirus* genus which includes Cache Valley and Kairi viruses (CVV and KRIV, respectively). This genus contains several emerging and re-emerging members that have caused disease in farmed livestock including Akabane virus (AKAV) in Africa and Asia, and Schmallenberg virus (SBV) in Europe [2–5]. CVV was first isolated from *Culiseta inornata* mosquitoes in Utah, USA in 1956 and has been detected in serosurveys in farm animals throughout North and Central America [6–14]. It has also been isolated from various other culicine and anopheline mosquitoes, including *Aedes (Ae*.*) sollicitans*, *Ae*. *vexans*, *Ae*. *cinereus*, *Ae*. *albopictus*, *Anopheles (An*.*) punctipennis*, *An*. *quadrimaculatus*, *Coquillettidia perturbans*, *Mansonia titillans*, *Culex (Cx)*. *salinarius*, several *Ochlerotatus* species, and *Psorophora columbiae* in the U.S., Canada and Mexico [9,15–21]. Sheep are particularly affected and CVV causes abortions or congenital malformations in pregnant ewes [22–26] as well as other disease symptoms [14]. CVV continues to increase its geographical range and was recently diagnosed in sheep in Ontario and Quebec although the virus was detected in Ontario much earlier in 1977 [27–29]. This virus has also been detected in serosurveys of humans and has been linked to several cases of sometimes fatal meningitis and encephalitis [15,30–33]. Moreover different lineages of CVV are beginning to emerge and a subtype, Maguari virus (MAGV), is also associated with disease in humans [34,35]. KRIV, like CVV, belongs to the Bunyamwera serogroup. It is another example of a potentially emerging virus of the Americas and was first isolated from various mosquito species in Trinidad including *Aedes*, *Wyeomia*, *Culex* and *Psorophora* ssp. [36]. It has also been isolated from mosquitoes and vertebrates in Central and South America, including from a febrile horse in Argentina [37–39]. In one serosurvey, antibodies (Abs) were identified in 6–18% of humans and up to 48% of horses [7,40,41]. KRIV does not cause any documented clinical disease symptoms in humans or animals.

Although the geographical ranges of CVV and KRIV, in North and South America respectively are mostly distinct (though MAGV has been detected in South America), they have both been isolated from the Yucatan peninsula of Mexico along with the closely related virus, Cholul (CHLV), which was suggested to be a reassortant of CVV and the related virus Potosi (POTV) [7,42]. POTV itself was suggested to be a reassortment of CVV and KRIV or a closely related virus [43]. Although this shared host range is limited, it may change and suggests the potential for reassortment between CVV and KRIV as well as other or yet unknown orthobunyaviruses to generate novel viruses. This is relevant to consider in vaccine design and vaccination studies, as vaccines could reassort with naturally circulating, related viruses.

Among the unifying characteristics of the previous family *Bunyaviridae* and the vast majority of the new order *Bunyavirales* members is the possession of a tri-segmented single-stranded genome of negative or ambi-sense polarity that encodes four structural proteins. The three genome segments (called L [large], M [medium] and S [small]) are encapsidated by the nucleocapsid (N) protein and are associated with the viral RNA-dependent RNA polymerase, the L protein, to form ribonucleoprotein complexes (RNP) termed nucleocapsids. RNPs are contained within a lipid envelope also containing the viral glycoproteins, Gn and Gc. Virus replication occurs in the cytoplasm of infected cells, and viruses mature primarily by budding from Golgi membranes. As well as the four structural proteins (L, Gn, Gc and N) many bunyaviruses encode two non-structural proteins, termed NSs and NSm. Genetic and biochemical analyses have shown that the S RNA segment encodes the N and NSs proteins; the M RNA segment encodes Gn, Gc and NSm as a polyprotein precursor; and the L RNA segment encodes the L protein [44–50]. The NSs proteins have been linked to viral virulence [51]. Reverse genetics systems for several orthobunyaviruses including Bunyamwera virus (BUNV), SBV, AKAV and La Crosse virus (LACV) have been developed over the last few decades [52–56] and studies on these viruses have increased our understanding of these pathogens. More importantly the use of reverse genetics systems of orthobunyaviruses have been used extensively to show that NSs proteins are important antagonists of type I interferon (IFN) responses [54,55,57–65]. Engineered orthobunyaviruses that do not express NSs or NSm proteins have also been assessed as candidate live vaccines for SBV [66].

The original protocol for BUNV reverse genetics was significantly improved by use of the T7 RNA polymerase-expressing cell line BSR-T7/5 [67,68]. This allowed recovery of BUNV directly from the transfected cells without a subsequent insect cell passage. It was also found that the antigenomic plasmids provided low levels of support proteins which were sufficient for an efficient rescue even in absence of support plasmids expressing L and N proteins [68]. Thus, recombinant BUNV can be generated just from antigenomic RNA. Here we have established minigenome and reverse genetics systems for CVV and KRIV and showed that deletion of NSs leads to attenuation and viruses that potently induce type I interferon responses. As CVV and KRIV overlap geographically in some areas but are phylogenetically distant we also assessed whether these viruses can reassort, by reverse genetics. No reassortants at least for the M segment were obtained; and vaccination with attenuated CVV in areas of shared geographical range is unlikely to result in reassortment between a CVV vaccine candidate and KRIV at least for this segment. This is important information to assess the environmental risk associated with vaccination.

## Results

### Sequencing and cloning of KRIV and CVV segments

To develop reverse genetic systems for CVV and KRIV, viral segments had to be cloned and this required accurate sequence data. We used the CVV strain 6V633, for which no sequence information was available at the time of cloning although an almost complete sequence was available for another strain (MNZ-92011). More recently sequence information was submitted to Genbank for the CVV strain 6V633; see Table 1. For KRIV we used strain TR8900, obtained by R. M. Elliott (an older designation for prototype KRIV TRVL8900; to distinguish virus used in this study, we will refer to our isolate as TR8900 and TRVL8900 for other published KRIV strain data). GenBank contained partial or full sequence for the S, M and L segments of different strains (Mex 07 and BeAr8226) though only partial information for KRIV TRVL8900 including a previously described S segment sequence for KRIV (strain not being specified in the supporting paper [69] but from records is likely to be TR8900; GenBank, accession number X73467.1) which was generated by using consensus primers for the S segment termini. These sequences were used to design gene specific primers for RACE analysis to amplify the full 5’ and 3’ untranslated regions (UTRs) to obtain the precise sequence of the 5’ and 3’ ends for all 3 segments. This allowed the design of RT-PCR primers to amplify and clone cDNA of the antigenomic RNAs for all 3 segments of both viruses. All segments for L, M and S antigenomes were cloned into pTVT7R [70], and sequenced. The coding sequences for L and N (which include the NSs open reading frame [ORF]), were also subcloned into the expression plasmid pTM1 for both viruses [71].

**Table 1:** Sequence coverage for KRIV (strain TR8900) and CVV (strain 6V633).

Segment	Reference sequence		New sequence	
	Accession no	Available sequence (nucleotide)	Accession no	Available sequence (whole sequence)
KRIV S (TRVL8900)	X73467.1* MH484302	1-992 1-990	MH166874	1-992
KRIV M (TRVL8900)	EU004186.1 MH484301	20-4586 2-4591	MH166875	1-4600
KRIV L (TRVL8900)	EU004191.1 MH484300	8-594 8-6899	MH166876	1-6910
CVV S (6V633)	KX100133.1	1-950	MH166877	1-950
CVV M (6V633)	KX100134.1	1-4463	MH166878	1-4463
CVV L (6V633)	KX100135.1	1-6870	MH166879	1-6870

The table shows the sequence coverage and accession numbers of reference sequences present in GenBank, and new consensus sequences generated by this project which have been deposited in this database. Nucleotide numbers of reference sequences refer to positions following alignment to sequences obtained here.

* from analysis of records this strain is likely to be TRVL8900.

To confirm sequence data obtained following cloning and sequencing, next generation sequencing (NGS) was performed on total cell RNA from BHK-21 cells infected with wild type (wt) CVV as described in the Methods. Results were obtained for CVV (read depth averaging 337 to 1695 reads/nucleotide; and with > 99.4% coverage). For KRIV, two separate batches of viral RNA from supernatant of approximately 20 ml and 50 ml from infected BHK-21 cells were used for NGS analysis. Results were obtained for KRIV (read depth >8836 reads/nucleotide; and >99.9% coverage), and we compiled consensus sequences for all three segments for both viruses with the information above (NGS and cDNA sequences); see GenBank accession below (Table 1). Full NGS data is available at the European Nucleotide Archive (ENA) with accession number PRJEB25770.

A comparison of sequencing data was carried out to assess our consensus sequences for all segments. For CVV three differences were noted between the recently available GenBank reference for strain 6V633 and our NGS and cDNA sequence data (Table 2); occasional mismatches at the extreme 3’ and 5’ termini following NGS were discounted. Two differences resulted in amino acid changes; one each in the M segment polyprotein and L protein. In another instance our sequence data of cloned virus-derived cDNA differed from the NGS and the reference sequence for a nucleotide in the 3’ UTR region of the antigenome of the CVV M segment. This was likely an error introduced when cloning the CVV M segment and the NGS and reference strain sequence are in all likelihood the correct sequences. This mutation was then corrected in our CVV M segment cDNA clone for virus rescue.

**Table 2:** Nucleotide and amino acid sequence comparisons.

Segment	Nucleotide				Amino acid change		
	position	reference	cDNA	NGS	position	reference	sequencing
KRIV S	706	G	T	T	209	Alanine (A)	Serine (S)
KRIV S	749	A	G	G	223	Lysine (K)	Arginine (R)
KRIV M	62	C	T	T	-	-	-
KRIV M	3034	C	T	T	964	Serine (S)	Serine (S)
KRIV M	3962	G	A	A	1274	Glycine (G)	Serine (S)
KRIV L	14	T	C	C	-	-	-
CVV M	2094	T	G	G	682	Asparagine (N)	Lysine (K)
CVV M	4448	T	C*	T	-	-	-
CVV L	5963	C	G	G	1972	Threonine (T)	Serine (S)

The table highlights differences between reference sequences for CVV (strain 6V633) and KRIV (strain TRVL8900) present previously in GenBank, and sequences obtained by NGS, or RACE analysis/sequencing of cloned segments (“cDNA”) for the virus strains used in this study, CVV (strain 6V633) and KRIV (strain TR8900). For clarity, amino acid positions noted in the M segments are for the polyprotein. Accession numbers of reference and consensus sequences for viruses used in this study are listed in Table 1.

* mutation identified only in the cloned antigenome cDNA; amino acid sequence derived from reference and consensus sequences as defined above. The GenBank reference sequences used for comparison have the following accession numbers: (KRIV TRVL8900) X73467.1, EU004186.1 and EU004191.1; (CVV 6V633) KX100133.1, KX100134.1 and KX100135.1.

Comparing our consensus sequence data for KRIV (strain TR8900) to the partial reference sequences available for the prototype TRVL8900 strain in GenBank, six nucleotide differences were found (Table 2). Two of the differences were in the non-coding regions whereas three other differences resulted in amino acid changes- two in nucleoprotein N and one amino acid change in the M segment polyprotein. The final nucleotide difference in KRIV M segment resulted in a synonymous codon in the polyprotein coding sequence. The different sequence data sets we generated by NGS and RACE analysis/sequencing of cloned segments were in complete agreement for KRIV (again mismatches at the extreme 3’ and 5’ termini following NGS were discounted). Additional sequences for KRIV segments (strain TRVL8900), but mostly missing the extreme termini (with the following accession numbers: MH484302, MH484301, MH484300; see Table 1) were, apart from the missing termini, identical to those described here.

**Fig 1:**
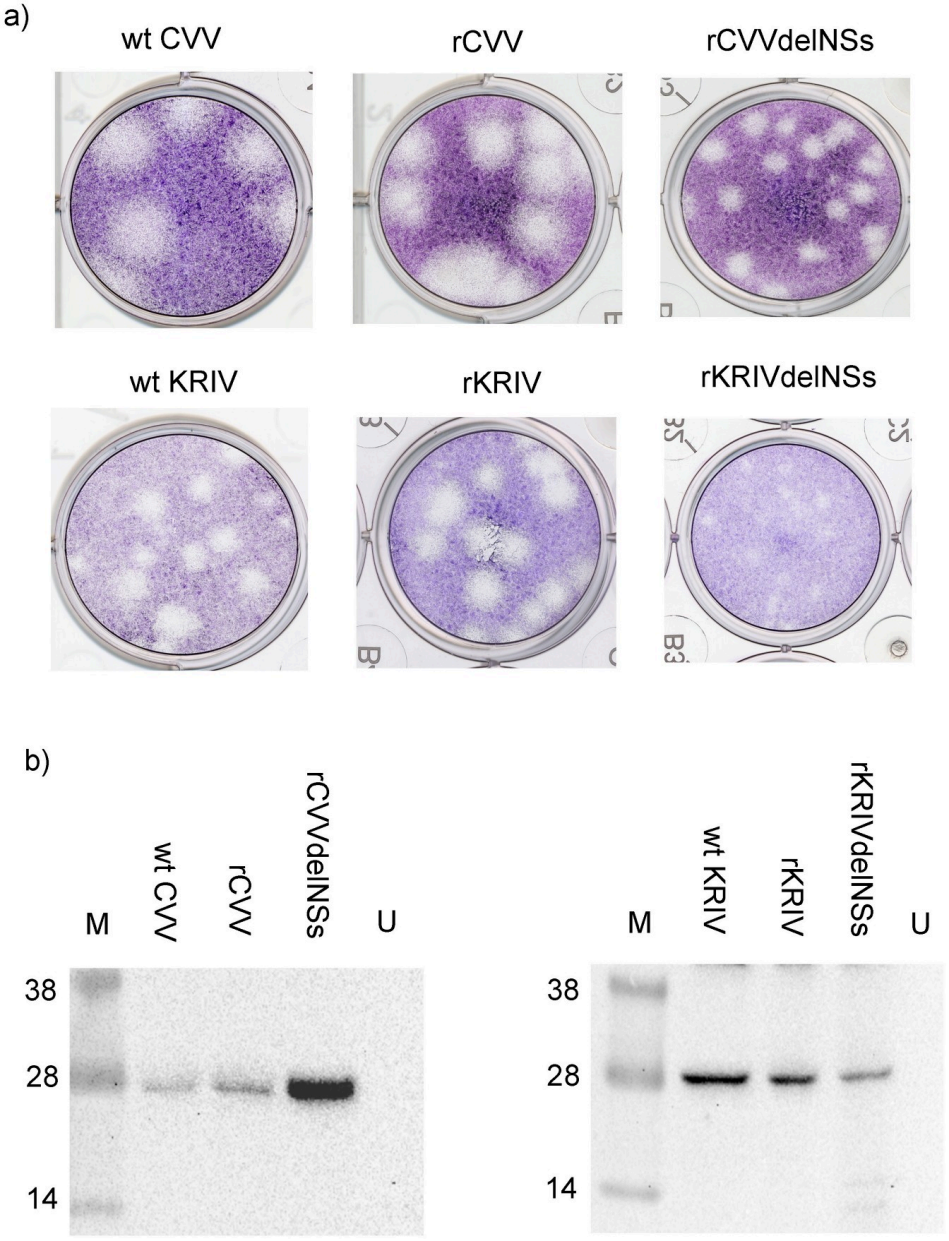
Growth of CVV and KRIV. **(a)** Plaque phenotype of authentic wt CVV and KRIV denoted as wt CVV and wt KRIV; recombinant wt CVV and KRIV, denoted as rCVV and rKRIV and NSs-deletant CVV and KRIV, denoted as rCVVdelNSs and rKRIVdelNSs. BHK-21 cells were used in all plaque assays. The plaque pictures shown are representative of several plaque assays. **(b)** Western blot analysis showing reactivity of infected BHK-21 cell lysate with anti-KRIV N and anti-CVV N antibodies. Molecular weight markers (M) in kD. The blot shown is a representative of 3 experiments with identical results; U, uninfected cells.

For CVV, pTVT7R-based plasmids containing the full length antigenomic cDNAs, were used in a three-plasmid rescue system, by transfection into the T7 RNA polymerase-expressing BSR-T7/5 cells without the use of additional expression plasmids for L and N proteins. The supernatant was harvested after 3–4 days, with highly visible cytopathic effect (CPE) and the virus was titrated by plaque assay. Similarly, for KRIV plasmids containing the full length antigenomic cDNAs, cloned into pTVT7R, were used in a five-plasmid rescue system. This included the use of expression plasmids (pTM1-KRIVL and pTM1-KRIVN) for the L and N proteins as described previously [68]. The supernatant media was harvested after 6 days, when CPE was visible, and the virus titrated in a plaque assay. The rescue of both viruses was repeated twice to assess robustness of the assay. The plaque morphology of the recombinant wild type viruses, designated rKRIV and rCVV, was indistinguishable from that of the authentic wt viruses (Fig 1).

**Fig 2:**
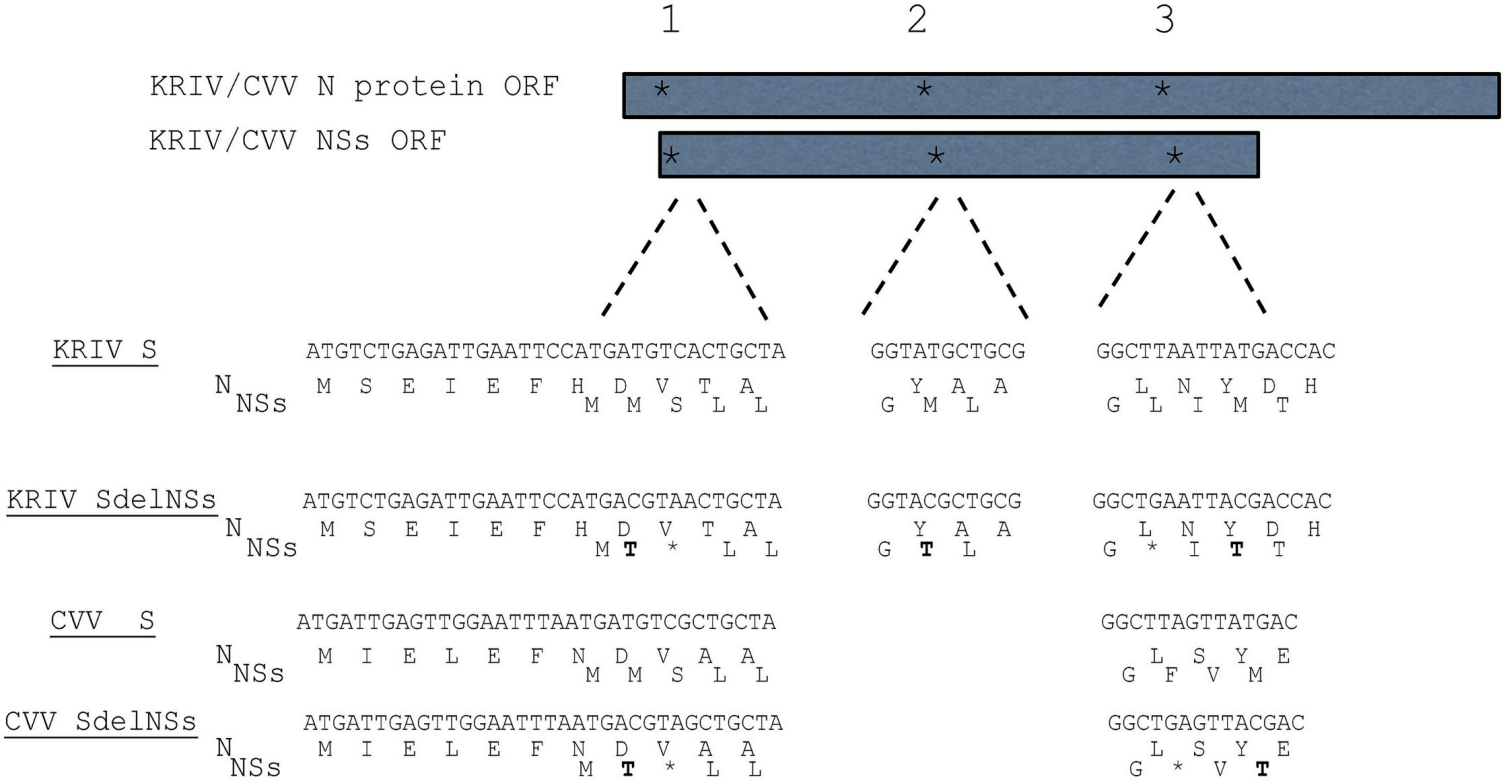
Design of CVV and KRIV that do not express NSs. Shown are sections of S segment for the N-termini of the N and overlapping NSs proteins. Mutations were added to disrupt the reading frames of the NSs proteins for both viruses, without changing the amino acid sequence of the overlapping N protein. For CVV, two methionines were changed to threonine (denoted in bold) and two stop codons introduced (denoted with an asterisk). For KRIV, mutational sites were used where three methionines were changed to threonines and two stop codons introduced. Protein representation is not to scale.

To investigate the function of the NSs proteins of both viruses, mutations were introduced into the NSs ORFs to produce recombinant viruses that no longer expressed this protein. These mutations were introduced in such a way as to prevent amino acid changes in the overlapping N protein ORF (Fig 2). Viruses denoted by rCVVdelNSs and rKRIVdelNSs were rescued as above by substituting the plasmids containing wt S segments with those containing mutations in the NSs genes. Nucleotide sequences of recovered viruses were determined by RT-PCR and sequencing except for the extreme termini, which were only sequenced by RACE for S of rCVV and rCVVdelNSs (see below). All sequences obtained matched those of the parental plasmids and mutations in the S segments of recombinant rCVVdelNSs and rKRIVdelNSs were confirmed.

The identities of the wt and recombinant viruses were also confirmed by western blotting of lysates of infected BHK-21 cells using polyclonal Abs raised against peptide regions of the N proteins (Fig 1b). We observed N levels consistently higher in rCVVdelNSs-infected cells compared to wt CVV and rCVV. As we did not find any mutations (including termini, as determined by RACE analysis of rCVVdelNSs, and for completeness also rCVV S segments) that could explain these observations, we speculate that removal of the NSs ORF is sufficient to lead to higher levels of N.

**Fig 3:**
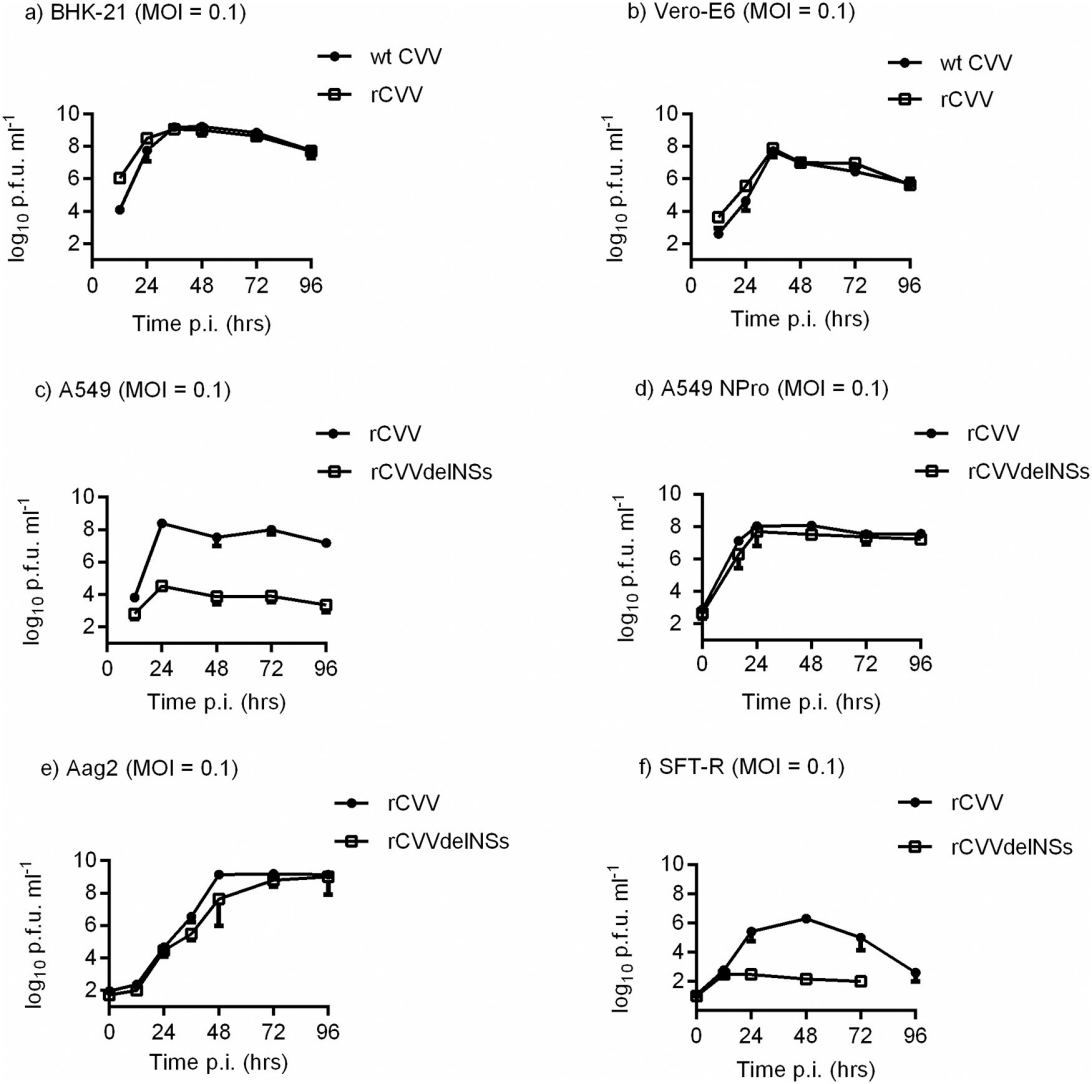
CVV growth in cell culture. Cell lines BHK-21 **(a)**, Vero-E6 **(b)**, A549 **(c)**, A549 NPro **(d)**, Aag2 **(e)** and SFT-R **(f)** were infected at a MOI of 0.1. The curves show the titre of CVV accumulated at 12 or 24 hr intervals. The titre of the virus at each time point was determined by plaque assay; virus titres are indicated in plaque forming units per ml (PFU/ml). Representative experiments are shown (n = 2). Error bars indicate standard deviation (SD).

**Fig 4:**
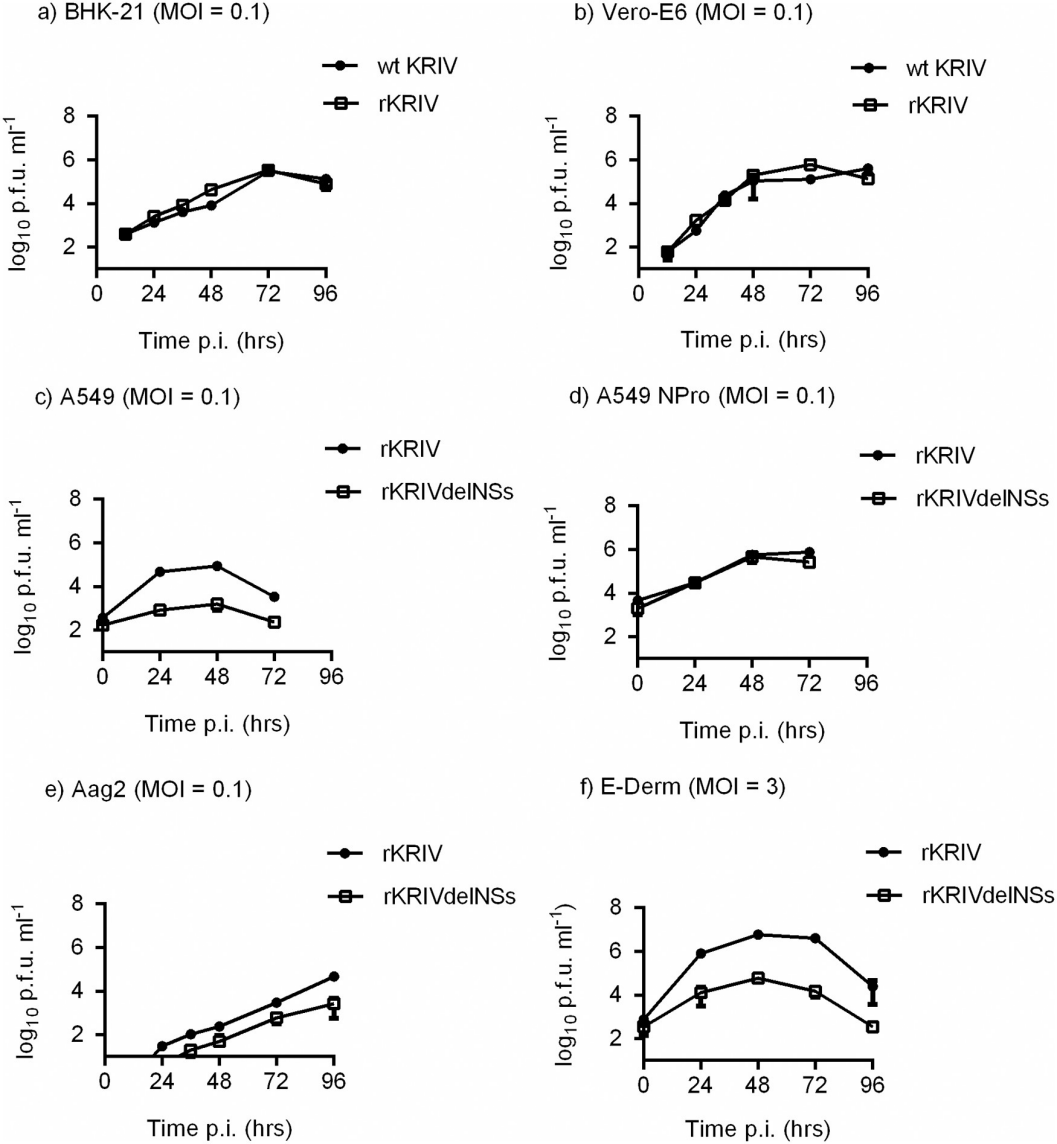
KRIV growth in cell culture. Cell lines BHK-21 **(a)**, Vero-E6 **(b)** A549 **(c)**, A549 Npro **(d)** Aag-2 **(e)** and the equine cell line, E-Derm **(f)** were infected at a MOI of 0.1 or 3 as indicated. The curves show the titre of KRIV accumulated after 12 or 24 hr intervals. The titre of virus at each time point was determined by a plaque assay; virus titres are indicated in PFU/ml. Representative experiments are shown (n = 2). Error bars indicate SD.

Growth of wt and recombinant CVV and KRIV were compared in BHK-21 and Vero-E6 cells infected at a multiplicity of infection (MOI) of 0.1 (Figs 3 and 4). No differences were observed in growth kinetics between wt and recombinant viruses in the two cell lines. Growth of the recombinant viruses rCVV and rKRIV and the NSs deletant rCVVdelNSs and rKRIVdelNSs were compared in the type I IFN competent A549 cell line with growth in A549 NPro cells, that express BVDV NPro protein which inhibits type 1 IFN production [72]. rCVVdelNSs displayed slower growth kinetics in A549 cells than rCVV, although in A549 NPro cells the two viruses displayed identical growth kinetics. Similar growth patterns were observed for rKRIV and rKRIVdelNSs in A549 and A549 NPro cells. Sheep are the main affected livestock species therefore the growth of rCVV and rCVVdelNSs were also compared in the ovine cell line SFT-R [66]. rCVVdelNSs displayed lower replication in SFT-R cells compared to rCVV. Due to the significant infection of the economically important horse species the growth of rKRIV and rKRIVdelNSs were also compared in the equine cell line E-Derm (NBL-6), following infection with a MOI of 3. Both recombinant viruses showed replication, with lower growth for rKRIVdelNSs (Fig 4). Since CVV and KRIV are both transmitted by mosquitoes, we also studied replication of these viruses in a commonly used mosquito cell line. Growth of the recombinant viruses rCVV, rKRIV as well as rCVVdelNSs and rKRIVdelNSs were assessed in *Ae*. *aegypti*-derived Aag2 cells infected at a MOI of 0.1. rCVV and rCVVdelNSs productively infected Aag2 cells (Fig 3); both rKRIVdelNSs and rKRIV productively infected Aag2 cells though titres were low (Fig 4).

### Type I IFN induction in the absence of CVV and KRIV NSs

**Fig 5:**
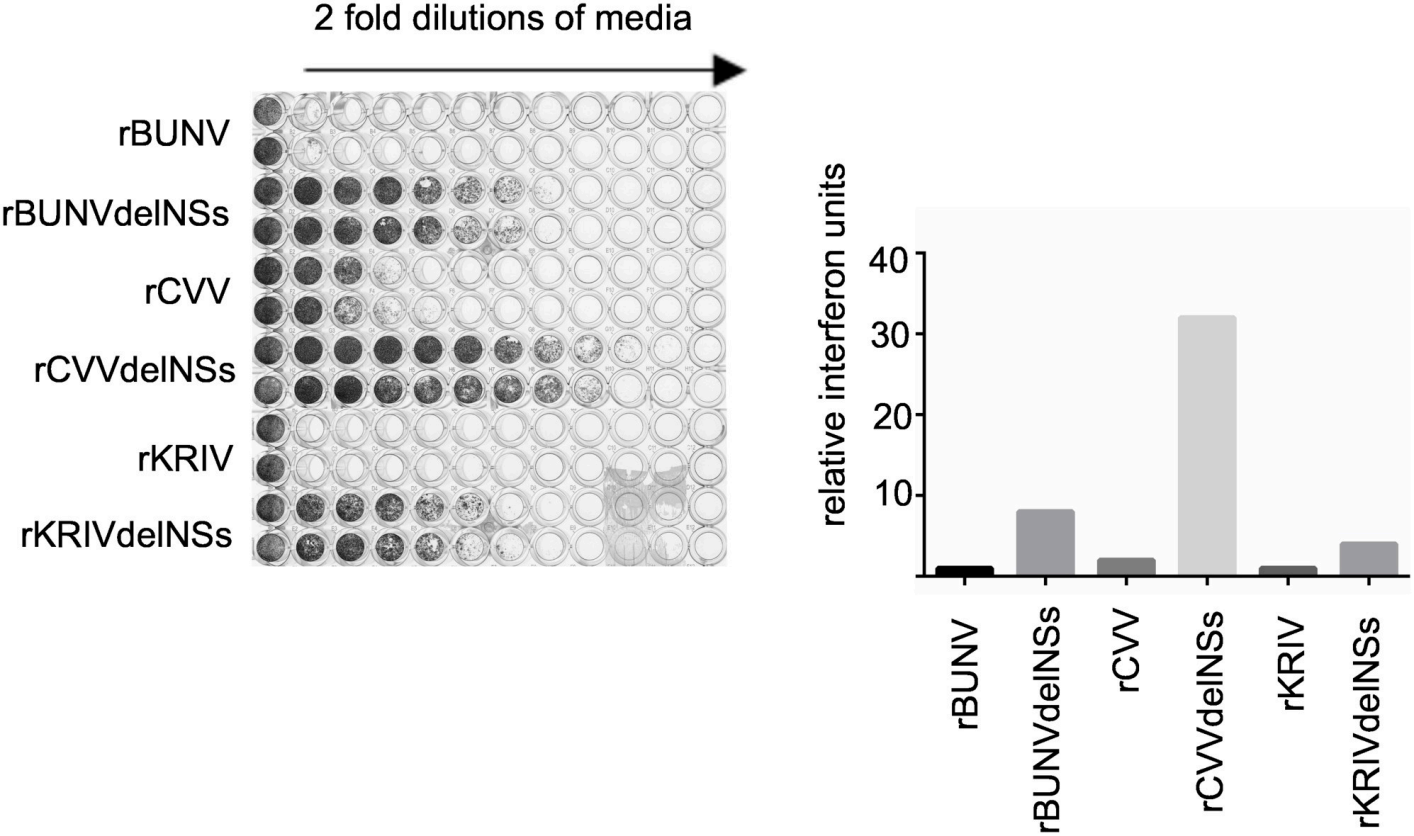
Type I IFN protection assay for orthobunyaviruses. A549 cells were infected with rBUNV, rCVV, rKRIV or rCVVdelNSs, rBUNVdelNSs (clone rBUNVdelNSs2) or rKRIVdelNSs at a MOI of 1 and incubated at 37 °C for 48 hrs. Twofold dilutions of the clarified and UV-treated supernatant were used to treat fresh A549 NPro cells in a 96-well plate for 24 hrs. The cells were then infected with EMCV and the development of CPE was monitored at 96 hrs post-infection by staining with crystal violet. The production of IFN was calculated according to the highest dilution of supernatant giving protection against EMCV infection and is expressed as relative IFN units. The experiment was conducted three times and gave identical results.

A biological assay used previously to monitor type I IFN production in response to orthobunyavirus infection was employed here to investigate the induction of type I interferon by rCVV, rKRIV, rCVVdelNSs and rKRIVdelNSs [73,74]. A549 cells were infected with recombinant viruses (MOI = 1) and following this, UV-inactivated medium from these cells was used to treat fresh A549 NPro cells which can respond to but cannot produce type I interferons. If present and active in the medium, type I IFN would induce an antiviral state and the cells would be protected from subsequent infection with a challenge virus, encephalomyocarditis virus (EMCV). Recombinant BUNV (rBUNV) and BUNV with a deleted NSs protein (rBUNVdelNSs2) were used as negative and positive controls, respectively [61]. The relative amounts of type I IFN produced were calculated according to the highest dilution of supernatant affording protection to the cells from EMCV infection as described in Methods. As shown in Fig 5, medium from A549 cells infected with wt viruses contained much less IFN than medium infected with their NSs-lacking counterparts. Medium from rCVVdelNSs, rKRIVdelNSs and rBUNVdelNSs2-infected cells largely protected A549 NPro cells from EMCV infection, indicating induction of IFN in the initial infections. Thus, the NSs proteins of CVV and KRIV suppress the production of type I IFN.

### Complementation and reassortment between CVV and KRIV

**Fig 6:**
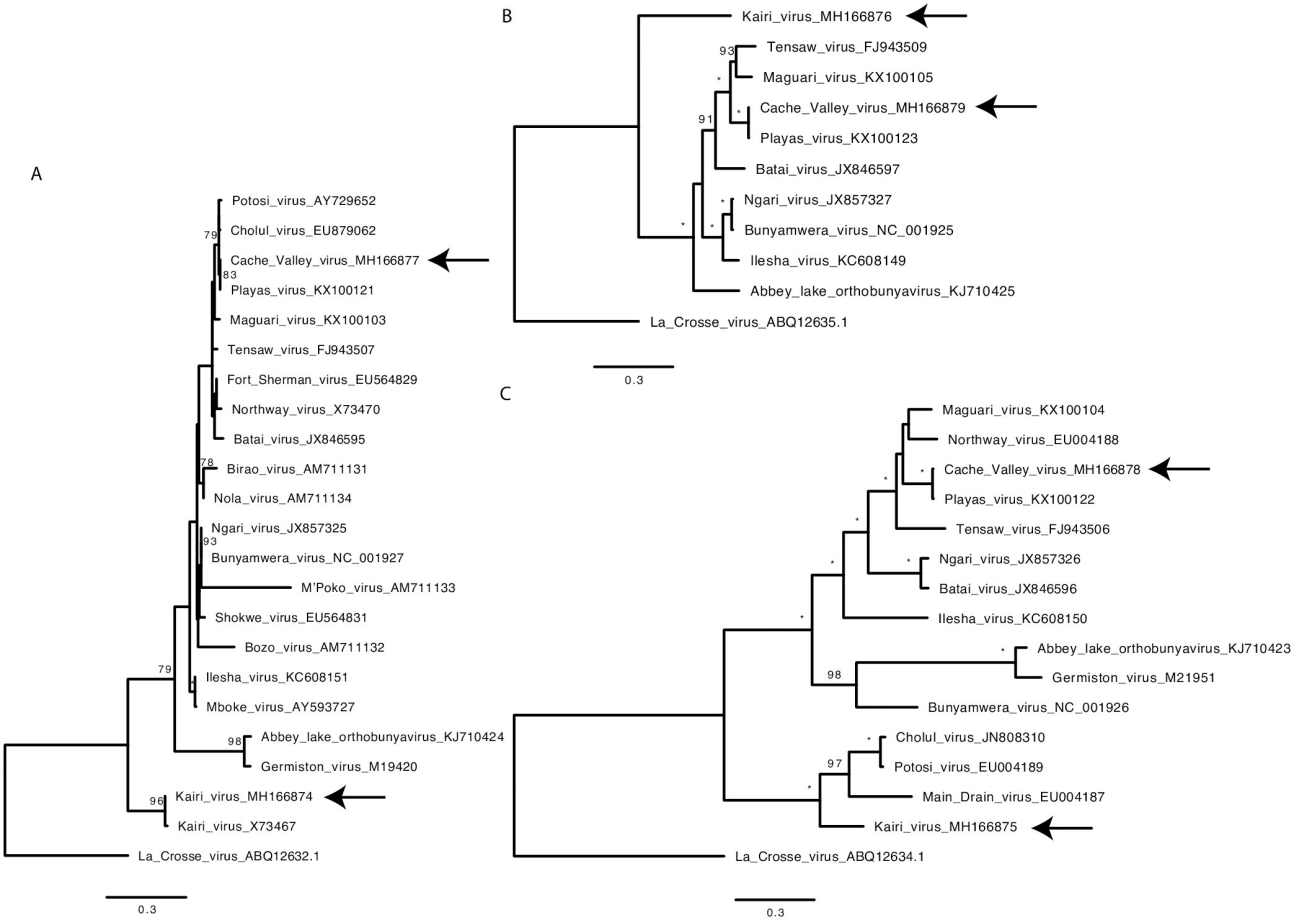
Phylogenetic analysis of selected viruses from the Bunyamwera serogroup with the newly acquired sequences from CVV (6V633) and KRIV (TR8900). The bootstrap supports shown at the nodes on the phylogeny (* represents 100 percent bootstrap support) and La Crosse virus (California serogroup) was used as an outgroup: **(A)** nucleoprotein N (from S segment) using the JTT+G substitution model, **(B)** RNA dependent RNA polymerase (from L segment) using the LG protein substitution model and **(C)** the M segment open reading frame (encoding glycoproteins and NSm) using the FLU+I+G+F substitution model. The arrows on the phylogeny indicate the phylogenetic position of the newly sequenced full genomes.

**Fig 7:**
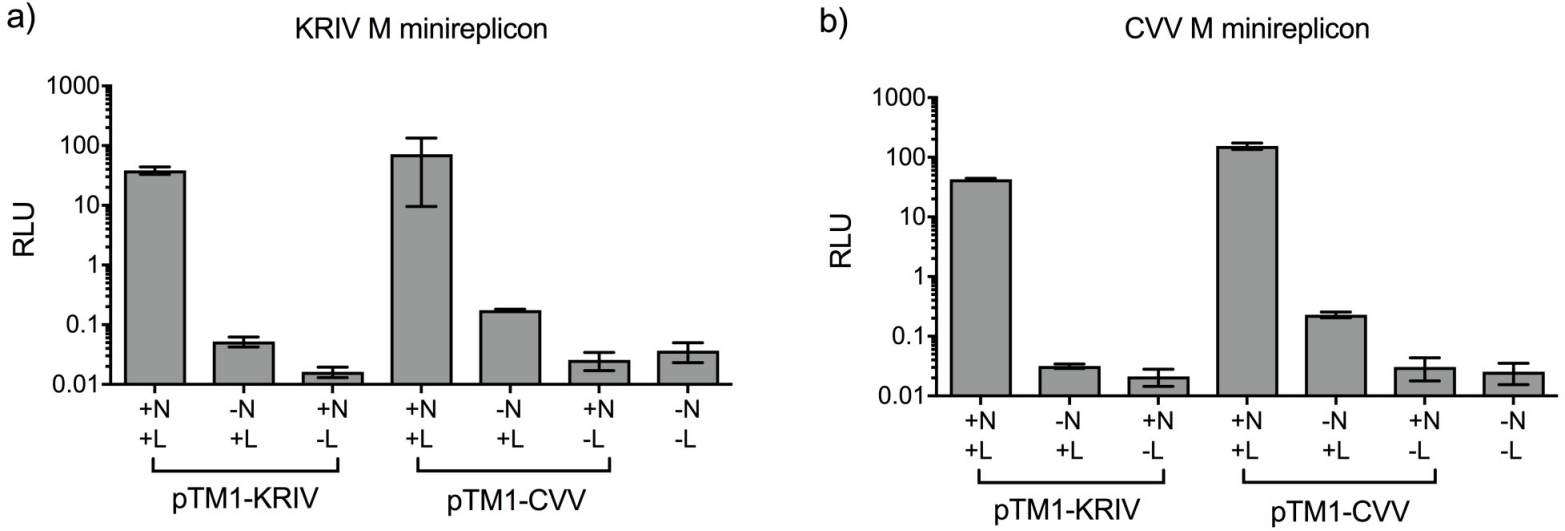
Cross-recognition of UTRs between CVV and KRIV in minigenome activity assay. BSR-T7/5 CL21 cells were transfected with pTM1-N and/or pTM1-L and M-derived minigenome plasmids (as described in Methods) pUC57-T7-KRIVMRen(-) **(a)**, or pUC57-T7-CVVMRen(-) **(b)**. Firefly luciferase (FF)-expressing pTM1-FF-Luc was used as a transfection control. At 24 hrs post-transfection, cells were lysed and Ren and Firefly luciferase activities were measured using Dual-Luciferase Reporter Assay kit (Promega). Luciferase values were normalised and minigenome activity is expressed as relative light units (RLU). Error bars indicate SD (n = 3). This is one representative experiment of three repeats with very similar results. pTM1-CVV or pTM1-KRIV refers to the viral origin of L or N proteins.

**Fig 8:**
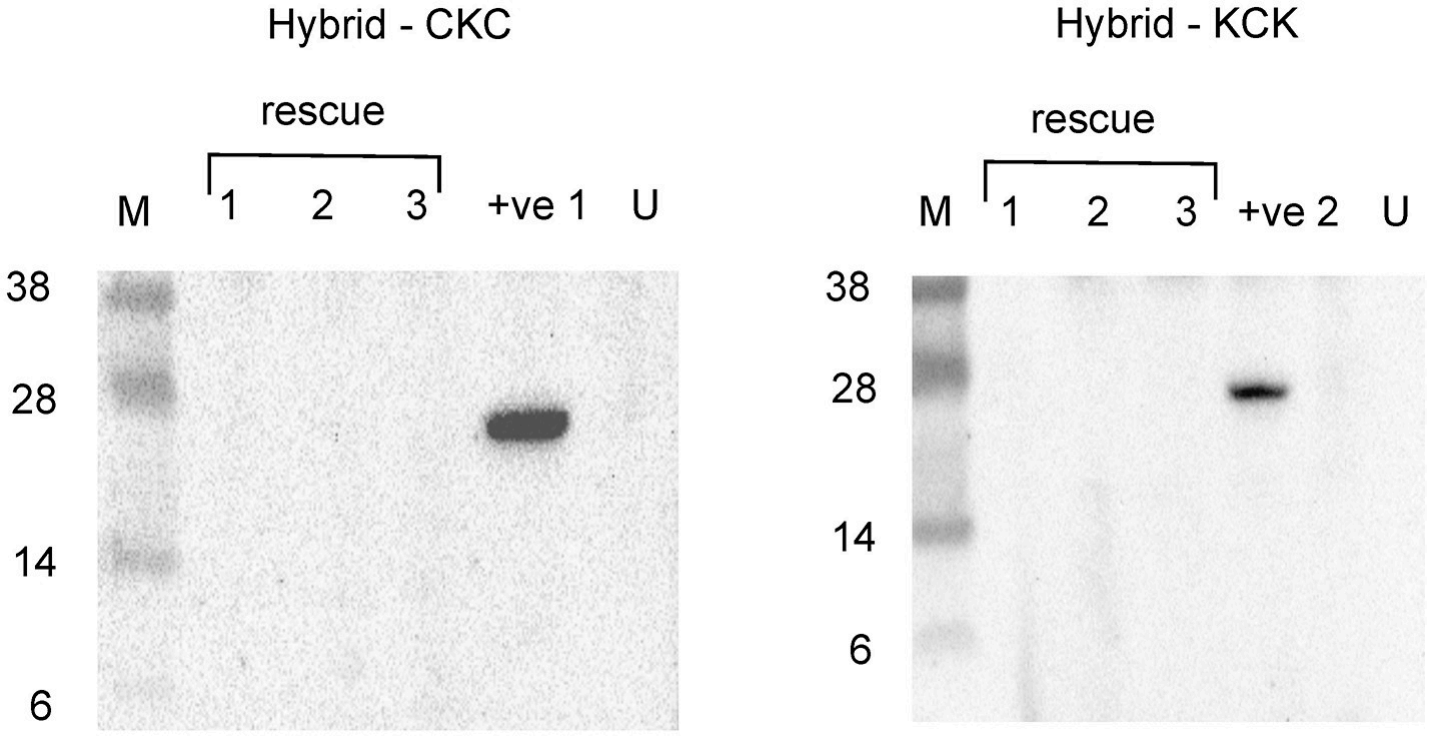
Attempted rescues of CVV and KRIV M segment reassortants. Western blot analysis showing the lack of N protein produced in 3 independent rescues for the hybrid viruses CKC and KCK (with segment order S-M-L). Supernatant from rescue experiment was passaged (1 ml of rescue supernatant added to BHK-21 cells for 5–7 days) and cell lysates used for western blotting using an anti-CVV N antibody for the hybrid CKC and an anti-KRIV N antibody for KCK. +ve 1, rCVV-infected cell lysate; +ve 2, rKRIV-infected cell lysate; U, uninfected cells.

Phylogenetic analysis of CVV and KRIV open reading frames as well as comparisons of nucleotide and amino acid identities indicate that CVV and KRIV are not closely related (Fig 6, Table 3). However as it is of interest to know whether such viruses could still reassort, for example post vaccination in areas of geographical overlap, we employed several genetic tools developed here. Previous work has shown the intracellular reconstitution of replication active BUNV nucleocapsids from transiently expressed components [75]. Here we developed similar minigenome systems for CVV and KRIV. The minimal components of this system are expression plasmids for L and N proteins and a minigenome; here an analogue of viral RNA supplied as a negative sense reporter gene (*Renilla* luciferase, Ren) cloned within the M genome segment UTRs and transcribed by T7 RNA polymerase from the corresponding promoter. The CVV-derived minigenome contained one change described above in Table 1 (position 4448 of the antigenome or 16 of the genome; the minigenome was active, as shown below). Plasmids were transfected into BSR-T7/5 CL21 cells and the results showed strong increase in luciferase levels above negative control levels (no expression of either N or L proteins, or both), demonstrating that we were able to reconstitute replication active CVV and KRIV nucleocapsids (Fig 7). In order to explore the reassortment potential of KRIV and CVV with each other, we also investigated if the minigenomes containing the M segment UTRs for both viruses could be swapped between the 2 minigenome systems. The results showed that for both CVV and KRIV, minigenomes are interchangeable and that functional nucleocapsids can be formed, at least with M-derived minigenomes (Fig 7). To assess if we could obtain hybrid viruses between CVV and KRIV, we used the 3 and 5 plasmid rescue system described above, and swapped the M segments because this is a frequent natural reassortment combination [76], though other reassortment combinations may be possible and were not assessed here. However, several attempts to rescue such hybrids, failed to produce viruses (Fig 8).

**Table 3:** Amino acid and nucleotide sequence identity comparisons across entire S, M, and L segments of selected orthobunyaviruses.

S segment					
% amino acid identity	% nucleotide identity				
	CVV	CHLV	MDV	POTV	KRIV
CVV		97.89	82.95	89.05	66.77
CHLV	99.15		82.74	89.16	66.57
MDV	89.74	89.74		82.74	66.87
POTV	99.15	99.15	89.74		66.37
KRIV	70.09	69.66	71.37	70.51	
M segment					
% amino acid identity	% nucleotide identity				
	CVV	CHLV	MDV	POTV	KRIV
CVV		59.00	59.13	59.41	59.06
CHLV	55.66		72.28	95.69	68.41
MDV	56.00	77.43		71.89	67.43
POTV	55.79	97.71	77.57		69.17
KRIV	56.49	76.44	72.29	77.07	
L segment					
% amino acid identity	% nucleotide identity				
	CVV	CHLV	MDV	POTV	KRIV
CVV		N/A	N/A	81.78	65.30
CHLV	N/A		N/A	N/A	N/A
MDV	N/A	N/A		N/A	N/A
POTV	93.30	N/A	N/A		65.33
KRIV	66.52	N/A	N/A	66.25	

Abbreviations and GenBank accession numbers for S, M, and L segments, respectively: CVV (MH166877, MH166878, MH166879); CHLV, Cholul virus (accession numbers EU879062.3, JN808310.1, N/A); MDV, Main Drain Virus (accession numbers X73469.1, EU004187.1, N/A); POTV, Potosi virus (accession numbers MF066370.1, MF066369.1, MF066368.1); KRIV (MH166874, MH166875, MH166876); N/A- sequence not available.

## Discussion

In this study we developed reverse genetics system for two orthobunyaviruses, CVV and KRIV, and used these to produce recombinant viruses (rCVVdelNSs and rKRIVdelNSs) that no longer express NSs. These viruses were attenuated across several cell lines and IFN protection assays confirmed the role of the KRIV and CVV NSs proteins as type I interferon antagonists in mammalian cells, as expected from previous studies [51]. CVV and KRIV did not require NSs to support replication in the mosquito cell line Aag2. This has also been observed for BUNV and SBV in the mosquito cell line C6/36 [53,77]. It would also be informative to test the growth of CVV and KRIV in other mosquito cell lines to further assess the role of species and immune status in replication. The production and characterisation of NSs-deletant, recombinant CVV is a first step towards developing an attenuated viral vaccine for this increasingly important livestock pathogen. Further attenuation could possibly be achieved by also deleting the NSm protein as described for SBV [66]. We also assessed the reassortment potential of CVV and KRIV. Indeed reassortment between orthobunyaviruses has been shown to be a key driver of orthobunyavirus evolution [43] and pathogenic viruses such as Ngari are reassortants of other known “parent” viruses, BUNV and Batai virus [43,78,79]. SBV is also a potential Shamonda/Sathuperi virus reassortant [80]. Indeed, POTV has been suggested to be a reassortant of CVV and KRIV, or a closely related virus [43]. In areas of overlap, vaccination with attenuated CVV could thus potentially lead to novel reassortants and should be considered in risk assessments. As CVV and KRIV L and N can replicate M segment derived minigenomes of either virus, we assessed compatibility of M segments between CVV and KRIV in virus rescues. This is the predominant segment that is acquired in novel hybrid orthobunyaviruses found in nature and evidence suggests that this is due to the interaction between N and L leading to linkage of L and S segments [76,81]; though viruses such as CHLV (likely a combination of the M and L segments from POTV and the S segment from CVV [42]) have been described. However, here we were unable to rescue the hybrid viruses containing alternate M segments. There could be several reasons for this; low efficiency of the rescue system, or that these hybrid viruses are unable to propagate. Several lines of evidence also suggest an interaction between both of the bunyavirus glycoprotein cytoplasmic tails and nucleocapsids, which could also influence the packaging of a hybrid virus [44,82].

In summary, we describe the generation of rescue systems for both CVV and KRIV and use these to demonstrate that the deletion of NSs leads to attenuated viruses no longer able to inhibit type I interferon responses. An attenuated CVV or KRIV containing a deleted NSs gene could serve as vaccine candidate. Additionally, reassortment by swapping CVV and KRIV M segments could not be demonstrated here by using reverse genetics. The poor conservation between CVV and KRIV M segments (Table 3 and Fig 6) may explain this observation. However other reassortment combinations can now be explored using reverse genetics systems, including those with more or less closely related orthobunyaviruses. We emphasize that reassortment by reverse genetics may have technical limitations and co-infection experiments or co-infections in nature may generate reassortants that are not obtained by such approaches, or that different plasmid combinations and concentrations used for reverse genetics may still generate other types of viral reassortants. Indeed, as discussed above, CHLV virus (M and L segments likely from POTV, S segment from CVV) [42] suggests that different reassortment outcomes are possible. Reverse genetics systems for CVV and KRIV, as well as other orthobunyaviruses, will be useful to test to what extent reassortment between these viruses is possible. To conclude, the systems developed here are relevant to further studies on orthobunyaviruses that include reassortment and vaccine design.

## Methods

### Cells

BHK-21 cells (provided by R. M. Elliott, University of Glasgow, UK) were grown in Glasgow’s minimal essential medium (GMEM) supplemented with 10% tryptose phosphate broth (TPB), 10% newborn calf serum (NBCS), 1000 units/ml penicillin and 1 mg/ml streptomycin (p/s). BSR-T7/5 cells, which stably express T7 RNA polymerase [67] were provided by K.-K. Conzelmann (Max-von-Pettenkofer Institute, Munich, Germany) and grown in GMEM supplemented with 10% TPB, 10% fetal calf serum (FCS), p/s and 0.25 mg/ml G418. These cells were used to rescue rCVV, rKRIV, rCVVdelNSs and rKRIVdelNSs. The BSR-T7/5 cells were also sub-cloned by dilution cloning to create a population of cells with a higher expression of T7 RNA polymerase; this new cell line developed by us was designated BSR-T7/5 CL21 [83] and used for minireplicon studies and rescues of virus reassortants. SFT-R cells (CCLV-RIE 43, Collection of Cell Lines in Veterinary Medicine [CCLV]), were obtained from the Friedrich-Loeffler-Institute, Greifswald-Insel Riems, Germany. These cells were grown in DMEM supplemented with 10% FCS and low glutamine (1g/L). E-Derm cells (also called NBL-6) (CCL-57, ATCC) were grown in DMEM containing 15% FBS and 1% NEAA. Vero-E6 cells were also provided by R. M. Elliott (University of Glasgow, UK). The A549 and A549 NPro cell lines were a kind gift from R. E. Randall (University of St. Andrews, UK). A549, A549 NPro and Vero-E6 cells were maintained in Dulbecco’s modified Eagle’s medium (DMEM) supplemented with 10% FCS and p/s. The A549 NPro cell media also contained 10 μg/ml blasticidin or 2 μg/ml puromycin as a selection agent. All mammalian cell lines were grown at 37 °C with 5% CO2. Aag2 cells were obtained from P. Eggleston (Keele University, UK) and grown in L-15 medium with 10% FBS, 10% tryptose phosphate broth and p/s. All media and reagents were purchased from Gibco, Life Technologies. BHK-21 cells were chosen to propagate and titre CVV and KRIV in plaque assays.

### Viruses

CVV (strain 6V633) and KRIV isolates (strain TR8900) were made available by R. M Elliott. The isolates were used to infect Vero-E6 cells and propagated for 5–6 days at 33 °C. Both viruses were then titrated by plaque assay, for initial characterisation studies on BHK-21 and Vero-E6 cells, under an overlay comprising MEM supplemented with 2% NBCS and 0.6% Avicel (FMC) and incubated at 37 °C for 3–5 days. Cell monolayers were fixed with 4% formaldehyde and plaques were visualized by staining with crystal violet staining solution. A working stock for both viruses was made by growing these in BHK-21 cells for 5–6 days at 33 °C at a MOI of 0.05. Recombinant viruses (rCVV, rCVVdelNSs, rKRIV and rKRIVdelNSs) were treated similarly, except rKRIVdelNSs which required 5–6 days to develop plaques on BHK-21 cells. Recombinant rBUNV and BUNV containing a deleted NSs protein (rBUNVdelNSs2) were used as controls in the IFN bioassay [53]. EMCV used in the IFN protection assay was obtained from R. E. Randall, University of St. Andrews, UK.

### Antibodies

Antibodies were produced in collaboration with Cambridge Research Biochemicals. Peptide regions of the N proteins of CVV and KRIV, that were different in sequence from each other, were selected to raise polyclonal Abs to distinguish between both viruses (http://www.discoveryantibodies.com/anti-cache-valley-virus-cvv-nucleocapsid-antibody) (catalogue number crb2005018), (http://www.discoveryantibodies.com/anti-kairi-virus-kriv-nucleocapsid-antibody) (catalogue number crb2005080).

### RNA isolation

Total cell RNA was isolated from BHK-21 cells infected with wt virus in 6 well plates or 25 cm^2^ flasks for recombinant viruses, and purified using Trizol according to the manufacturer’s instructions. Viral RNA was isolated from supernatant of infected BHK-21 cells grown in 150 cm^2^ flasks collected at 5 days post infection. Briefly, supernatant was first clarified by centrifugation at 4000 RPM for 10 min. Then virus was concentrated by ultracentrifugation at 26000 RPM for 90 min on 20% sucrose cushion in PBS. RNA from pelleted virus particles was isolated using Trizol as above.

### NGS sequencing

Samples were prepared using an Illumina TruSeq Stranded RNA kit. Paired end data was generated with 2x150bp on MiSeq for CVV. Initial quality assessment was done using FastQC (https://www.bioinformatics.babraham.ac.uk/projects/fastqc/). The sequencing adaptors and sequence reads with Phred quality score less than 33 were trimmed using Trim Galore (https://www.bioinformatics.babraham.ac.uk/projects/trim_galore/). Quality trimmed and cleaned sequences were mapped to the reference genome segments for CVV (KC436106.1, KC436107.1 and KC436108.1) using short read mapper Tanoti (http://www.bioinformatics.cvr.ac.uk/tanoti.php) and consensus sequences were generated using SAM2CONSENSUS (https://github.com/vbsreenu/Sam2Consensus). Assembly statistics including number of mapped reads, depth and breadth of coverage were generated by using the weeSAMv1.1 software package (https://github.com/josephhughes/Sequence-manipulation/blob/master/weeSAMv1.1). Two samples of KRIV were also sequenced using the MiSeq protocol and paired end data for the samples were analysed using the bioinformatics methods described above. However, Bowtie2 [84] was deemed to be a better aligner for the KRIV data and was used for the reference mapping. Short reads from KRIV samples were mapped to all segments by using cDNA (RACE/cloned segment) sequences obtained previously for the TR8900 strain and consensus was called using SAM2CONSENSUS. Default parameters were applied to all software packages used in this analysis.

### Cloning of CVV and KRIV antigenomic cDNAs and construction of delNSs S antigenomes

Supernatant (1 ml) from infected BHK-21 cells was concentrated in a Vivaspin 500 (Sartorius) and used to extract viral RNA (QIAamp viral RNA extraction kit). The viral RNA was used for RACE with a poly-A tailing Kit (Ambion) and standard PCR methods. The RNA was also used for first strand synthesis using primers designed from sequences derived from RACE analysis (Superscript III First Strand Synthesis System) (Thermofisher). All cDNAs were then amplified using Phusion high fidelity polymerase (NEB) purified, digested, and ligated into pTVT7R that had been linearized with BbsI [70]. The CVV S genomic segment was ligated into pTVT7R using BsmBI sites and the CVV M genomic segment was ligated into pTVT7R similarly using BfuAI sites also added to the 5’ and 3’ ends of the RT-PCR primers. The CVV L genomic segment was synthesized from a deep sequencing consensus, re-amplified and cloned into pTVT7R using the In-Fusion system. Resulting plasmids were named pTVT7R-CVVS, pTVT7R-CVVM and pTVT7R-CVVL. The KRIV S segment was cloned into pTVT7R using BsmBI sites that were added to both RT-PCR primers. The KRIV M and KRIV L genomic segments were ligated into pTVT7R using the In-Fusion system (Clontech). The plasmids were named pTVT7R-KRIVS, pTVT7R-KRIVM and pTVT7R-KRIVL. A G residue was added to the 5’ end of all cDNAs to increase transcription efficiency of the antigenome transcripts by T7 RNA polymerase. Viral RNA polymerase L and nucleocapsid N (containing the NSs ORF) protein expression constructs were also constructed by subcloning the cDNAs into the expression vector pTM1 [71]. This was achieved using PCR and the In-Fusion system (Clontech). The plasmids created were named pTM1-CVVL, pTM1-CVVN, pTM1-KRIVL and pTM1-KRIVN. All primers are available from the authors on request.

To stop expression of NSs expression by CVV S and KRIV S segments, start codons were removed and stop codons introduced by PCR mutagenesis using a method based on the Quikchange site-directed mutagenesis system (Stratagene). All mutations were designed to prevent amino acid mutation in the overlapping N proteins. For CVV NSs, 2 rounds of mutagenesis removed 2 start codons at amino acid positions 2 and 30 and introduced 2 stop codons at positions 3 and 28 in the NSs ORF to create the plasmid pTVT7R-CVVdelNSs (Fig 2). For KRIV NSs, 3 rounds of mutagenesis removed 3 start codons at amino acid positions 2, 17 and 30 and introduced 2 stop codons at positions 3 and 28 in the NSs ORF to create the plasmid pTVT7R-KRIVdelNSs (Fig 2).

### Rescue of CVV and KRIV viruses by reverse genetics

BSRT-7/5 cells (2x10^5^ per well) were seeded in 6 well plates and the media changed to GMEM supplemented with 10% TPB and 2% FCS on the day of transfection. Plasmids containing viral cDNAs (0.5 μg per plasmid) were transfected using Trans-IT LT-1 (Mirus) and the development of CPE monitored over 3 days. Supernatant was harvested after highly visible levels of CPE or after 6 days and titrated in a plaque assay to confirm the presence of rescued virus. For the attempted rescue of CVV and KRIV hybrid viruses, BSRT-7/5 CL21 cells (6x10^5^ per well) were seeded in small flasks (25 cm^2^). The media was changed to GMEM supplemented with 10% TPB and 2% FCS on the day of transfection. Plasmids containing viral cDNAs (1 μg per plasmid) were transfected using Trans-IT LT-1. The development of CPE was monitored after 3 days. After 6 days, with no visible CPE, supernatant was harvested and added to small flasks (containing BHK-21 cells) before testing for the presence of virus. 5 plasmid rescues were attempted using expression plasmids for N and L. Plaque assays were performed (staining with crystal violet), or western blotting of passaged virus using anti KRIV and anti CVV N antibodies.

### Minigenome constructs and replication assay

Minigenome reporter constructs were created by substituting the polyprotein sequence of the M genomic segment with the *Renilla* luciferase (Ren) gene. The constructs had in order the following components; T7 RNA polymerase promoter, 5’UTR of the genome, (Ren) luciferase (-ve sense), 3’UTR of the genome, hepatitis delta ribozyme and T7 RNA polymerase terminator. The resulting plasmids, designated pUC57-T7-CVVMRen(-) and pUC57-T7-KRIVMRen(-) were synthesized by Genscript.

BSR-T7/5 CL21 cells (1.5x10^5^ per well) in 12-well plates were co-transfected with 500 ng each of pTM1-based L and N protein expression plasmids, and M-based reporter plasmids, 10 ng of pTM1-FF-Luc as a transfection control, and appropriate amount of empty pTM1 vector to equalise total amounts of plasmid DNA; using 1.5 μl of Trans-IT LT1 per reaction. *Renilla* and Firefly luciferase activities were measured at 24 hrs post-transfection using a Dual-Luciferase Assay kit (Promega) according to the manufacturer’s protocol.

### Biological assay for IFN production

This assay was carried out essentially as described [73]. In brief A549 cells (5x10^4^ per well), grown in a 24 well plate, were infected with the different viruses as indicated at an MOI of 1 and incubated at 37 °C for 48 h. The supernatant fluid was clarified by centrifugation and residual virus inactivated by UV treatment. Thereafter twofold serial dilutions of the medium were applied to fresh A549 NPro cells grown in 96-well plates for 24 hrs. The cells were then infected with EMCV, which is sensitive to IFN, and incubated for 4 days at 37 °C. Cells were then fixed with formaldehyde and stained with crystal violet to monitor the development of CPE. The production of IFN was calculated according to the highest dilution of supernatant giving protection against EMCV infection and is expressed as relative IFN units.

### Phylogenetic analysis

The newly sequenced full genomes of CVV (6V633) and Kairi virus (TR8900) were collated with sequences from GenBank. The protein sequences were aligned using MAFFT [85] and the best substitution model was selected using ProtTest [86] using the Bayesian Information Criterion (BIC). A maximum likelihood tree was reconstructed using the best substitution model with RAxML [87] using 1000 bootstrap replicates for node support.

### Data link

Data for Figs 3, 4 and 7 are available under http://dx.doi.org/10.5525/gla.researchdata.599 while database accession numbers for sequencing data are available as indicated above.
